# Science policies and practice of including and excluding women from clinical studies: focus on cardiovascular research

**DOI:** 10.3389/fphys.2026.1776903

**Published:** 2026-05-08

**Authors:** Anton Kovalsky, Irena Levitan

**Affiliations:** 1 Science Advocacy and Writing group (SAW), Science Writing Group, Cary, NC, United States; 2 Division of Pulmonary and Critical Care, University of Illinois at Chicago, Chicago, IL, United States

**Keywords:** Belmont report, bioethics, cardiovascular clinical trials, gender inequities, public law

## Abstract

The under-representation of women in clinical studies remains a major issue, recognized by researchers, patients, and legislators alike. In this review, we examine the key legislative documents that have shaped policies governing the exclusion and inclusion of women in clinical research over the past several decades, and their impact on seminal cardiovascular studies. We focus on early federal human-subject regulations codified as the Common Rule, which initially contributed to the near-complete exclusion of women, and trace the gradual recognition of the need for women’s inclusion and the progress achieved to date. Key trials discussed include the Framingham Heart Study, the Multiple Risk Factor Intervention Trial (MRFIT), and the Physicians’ Health Study (PHS), as well as a meta-analysis of over 1, 000 cardiovascular trials conducted between 2017 and 2023. We also discuss the critiques of the legislation including the current reliance on informed consent from a bioethics perspective.

## Introduction

Ethical treatment of patients participating in biomedical studies is often defined as protecting patients’ rights, not subjecting them to any undue harm, providing informed consent, and keeping their information confidential. In the United States, legislation codifying such protections was passed into law in the 1970s. While these laws implemented critical requirements around risk and consent, they also resulted in some unintended consequences, excluding certain populations from participating as research subjects, leading to underrepresentation in trial data, and ultimately harming equity in implementation of medical interventions. This review interrogates how detrimental exclusionary practices have historically been a byproduct of necessary but poorly designed risk-mitigating regulations. Special attention is given to landmark studies in cardiovascular health, which revealed significant inequities in the realm of study and treatment of female patients. We further examine how these policies influenced the design of clinical studies and, consequently, the evidence base guiding cardiovascular care in women.

General Methodology: The sources were identified through targeted literature searches in PubMed using multiple key words related to the topic, reviewing historical policy documents from NIH and FDA repositories and cross-referencing the sources using prior reviews. Key policy documents were selected based on their impact on federal regulations and guidelines. Clinical studies were selected based on their recognition as landmark cardiovascular trials frequently cited in the literature and their relevance to the inclusion or exclusion of women. The intent was to highlight pivotal examples that influenced policy development and clinical practice rather than to provide an exhaustive or systematic survey of all studies.

## Common rule: historical restrictions on women in clinical trials

Federal protection of human subjects in the U.S. began to develop in the 1970s, in part due to harmful clinical trials internationally, such as those involving Thalidomide, a medication for morning sickness that caused severe birth defects. The National Research Act (Public Law 93-348) established a federal framework for the protection of human research subjects, which was subsequently implemented through regulations known as the Common Rule (45 CFR 46); these regulations define minimal risk as risk not greater than that ordinarily encountered in daily life (Williams, 2005). The Common Rule also introduced the requirement for informed consent, ensuring participants are provided a thorough explanation of the research, its risks, and its benefits. However, these early regulations led to restrictions on women’s participation in research. In particular, Subpart B of the Common Rule, which addresses additional protections for pregnant women, human fetuses, and neonates, introduced more stringent criteria for risk–benefit assessment and, in some cases, required the consent of both parents (US Department of Health and Human Services, O.f.H.R.P., 2025). While intended to enhance protection for women and unborn children, these provisions contributed to the broad exclusion of women from clinical research.

In 1977, the U.S. Food and Drug Administration (FDA) issued guidance titled *General Considerations for the Clinical Evaluation of Drugs*, which recommended caution in enrolling women of childbearing potential in early-stage clinical trials (Phase I and early Phase II) (Administration, U.S.F.a.D., 1977). This guidance was intended to prevent potential harmful fetal exposure, but it was applied broadly to all women of reproductive age, regardless of pregnancy status, contraceptive use, or sexual activity. While the regulations allowed women to participate in later-phase trials (Phase II and III) once initial safety was established, participation was stipulated to be conditional on a woman not becoming pregnant during the trial, which was difficult to predict and ethically problematic to enforce. As a result of these restrictive recommendations, many researchers interpreted the guidance as effectively requiring the exclusion of all women from early drug trials. This widespread exclusion had lasting implications for the evidence base, as early-phase studies inform dosing, safety, and mechanism of action, thereby shaping subsequent clinical development and therapeutic use.

## Belmont report: core ethical principles and critiques

The ethical principles formulated by the National Commission for the Protection of Human Subjects of Biomedical and Behavioral Research were summarized in the Belmont Report in 1979 (Research, U.S.N.C.f.t.P.o.H.S.o.B.a.B., 1979). The commission articulated three core principles: **respect for persons**, **beneficence**, and **justice**. The principle of respect for persons emphasizes recognition of individual autonomy, including the capacity to make informed decisions about participation in research.

Later ethical analyses have questioned some aspects of the Report. Kristinsson (2009) argued that placing the primary burden of risk assessment on individual participants is unfair, and that participants may give consent based on inaccurate information, poor comprehension, or subtle forms of coercion. In the biomedical context, coercion and deception may circumvent the participant’s agency, violating their rights. Because the Belmont Report does not explicitly state the moral value of informed consent but rather relies on its own authority to ascribe this value, ethicists like Kristinsson argue that such guidelines would better serve their purpose grounded in first principles of ethical analysis. Another later criticism held that despite tremendous improvement brought by the report’s guiding principles, it does not address the concerns of vulnerable communities, which cannot be sufficiently addressed by the principle of respect for persons (Friesen et al., 2017) due to inequities impacting those communities at a systemic level.

There was also a substantial ethical criticism of the Belmont Report around the absence of the feminist perspective, as it was pointed out that the concern for avoiding harm to women conflicts with the “rights” of women to participate in the studies and benefit from their findings (Medicine, I.o., 1994). It was also argued that distributive justice, the principle of fair allocation of society’s benefits and burdens, as formulated in the Belmont Report, does not address the systemic disadvantage of women and the hierarchical structure of our society.

It is critical to realize, however, that the Belmont Report emerged in response to well-documented ethical failures in human subjects research and at the time, the emphasis on minimizing risk and strengthening informed consent was both necessary and widely regarded as a critical safeguard against further harm. While, as described above, subsequent ethical analyses have identified some limitations of this framework, these critiques reflect the evolution of bioethical thinking in the decades following the Report’s publication and do not negate or minimize its value, as most significant step forward in addressing the issue.

## Considerations of excluding women beyond drug trials

It is important to note that protectionist policies do not account for excluding women from clinical studies that are not drug trials, as studies focusing exclusively on data gathering carry far less risk of harm, if any. The roots of this broader exclusion are widely argued to be based on sex and gender bias rather than protection. Notably, the exclusion of women went far beyond their exclusion from clinical drug trials and encompassed almost all biomedical studies. Several factors contributed to this phenomenon. First, was an assumption that male physiology is the standard human model, representative of both sexes, a concept that originated from biological determinism that viewed women as inherently inferior to men (Perry and Albee, 1998). Another factor was viewing the menstrual cycle and related hormonal fluctuations as “a complication”, which increases the complexity and cost of the study. Also, there was a belief that menstrual cycle renders women to be inherently more variable than men, even though later studies did not support this belief (Plevkova et al., 2020; Rocks et al., 2022). These considerations led to neglect of women in biomedical research with significant consequences for women’s health.

## Exclusion and under-representation of women in early seminal cardiovascular studies

The *Framingham Heart Study (FHS),* the seminal clinical study of cardiovascular risk factors that defined the field for decades was launched in 1948 and in contrast to many later studies included both men and women in almost equal proportion: the original cohort included 5, 209 patients with 45% men and 55% women (Kannel et al., 1961). An early report of the Framingham study unequivocally demonstrated significant sex differences in the development of coronary heart disease (CHD): the incidence of CHD in young women was >10-fold lower than in men with the gap decreasing to ~2-fold in the middle age (Kannel et al., 1961) but women with diabetes were shown to be uniquely vulnerable to the development of congestive heart failure (Kannel et al., 1974). A more detailed analysis of the sex-differences in the Framingham study cohort following a 26-year observation period found that women frequently exhibit silent myocardial infarctions and that unrecognized infarction is a significant problem requiring more frequent testing and particular attention to atypical symptoms (Lerner and Kannel, 1986). Clearly, it is imperative to include both men and women in clinical studies, yet several major studies as late as the 1990s either excluded women completely or strongly underrepresented them.

One of the first major large-cohort studies was the *Multiple Risk Factor Intervention Trial (MRFIT) (1973-82)* (Multiple Risk Factor Intervention Trial Research Group, 1982; Multiple Risk Factor Intervention Trial Research Group, 1997)*,* a randomized study with 12, 866 participants that tested whether counseling for smoking cessation and dietary reduction of cholesterol, together with more intense treatment for hypertension result in lowering the risk factors for and the mortality from CHD. Although the intervention was primarily counselling and not a drug trial, the study recruited men only, while women were excluded. Another men-only large cohort study of CHD prevention was *Physicians’ Health Study (PHS), 1980s* that tested the efficacy of aspirin for primary prevention against cardiovascular mortality (Hennekens and Eberlein, 1985). The study included 22, 071 physician males with the justification that they constitute a highly motivated and educated cohort that will show high compliance and precise results. The conclusions of the study were disappointing: while aspirin significantly reduced the incidents of a first MI, it did not reduce cardiovascular mortality and had a complication of increased hemorrhagic stroke. This conclusion was interpolated to women without taking into consideration possible sex differences. Another example is a landmark project called *Studies of Left Ventricular Dysfunction (SOLVD),* (late 1980s-early 1990s), which demonstrated the benefits of angiotensin-converting enzyme (ACE) inhibitors to treat chronic heart failure (Lam et al., 2020). The study included only a small proportion of women (~20%) and was strongly underpowered, failing to establish the efficacy of the treatment for women. Multiple other examples of such exclusion or strong underrepresentation of women in cardiovascular clinical trials include early studies of statins, thrombolysis, and coronary occlusion/reperfusion studies (Lee et al., 2001; Tsang et al., 2012).

Collectively, these examples illustrate how regulatory restrictions led negative clinical consequences for women health. The exclusion or underrepresentation of women resulted in clinical datasets that were insufficient to assess the prevalence, severity and disease progression that is specific for women, and as a result failed to adequately assess treatment efficacy and adverse effects. Moreover, the generalization of the findings from men to women led to incorrect assumptions, which contributed to weaker diagnosis and management of cardiovascular disease in women. It is also important to distinguish between studies that report *post-hoc* sex-stratified analyses and those that are prospectively designed and adequately powered to detect sex-specific differences. Subgroup analyses performed in underpowered cohorts can generate important hypotheses but are generally exploratory and cannot reliably establish sex-dependent effects. In contrast, trials that prospectively incorporate sex as a biological variable and are adequately powered for sex-specific analyses are required to generate robust evidence regarding sex differences in disease presentation, treatment response, and outcomes. This distinction remains highly relevant today, as many clinical trials include women but are not designed to detect sex-specific effects.

## Mounting criticism for the exclusion of women and policy changes

The issue of gender inequality began drawing increasing attention in the early 1980s, particularly regarding economic health. During a 1983 hearing of the Senate Committee on Finance on “Potential Inequities Affecting Women”, 1983, Senator Durenberger stated: “The disparity that exists between men and women in this country should shock the conscience of a nation founded on the principle of equal opportunity” (Senate, U.S., 1983). It can be argued that even though this hearing focused on correcting economic disparities via tax and retirement policies, it contributed to broader awareness of the disparities that existed between men and women, including in research participation.

More directly, the exclusion of women from clinical studies was addressed by the Public Health Service Task Force on Women’s Health Issues established in 1983 and led by Dr. Ruth Kirschstein, the first woman to lead a major NIH institute. A seminal report of the U.S. Public Health Service Task Force on Women’s Health Issues (1985) “Women’s health. Report of the Public Health Service Task Force on Women’s Health Issues” issued a recommendation that “Biomedical and behavioral research should be expanded to ensure emphasis on conditions and diseases unique to, or more prevalent in women in all age groups” (Force, U.S.P.H.S.T., 1985).

These recommendations and increased awareness led the NIH in 1986 to issue advisory guidance in the *NIH Guide for Grants and Contracts*, encouraging inclusion of women in clinical studies and requiring investigators to justify any exclusions (NIH, 1986). This was followed by FDA guidelines in 1988, which required demographic information including gender to be tabulated and reported, including benefits and risks for different subgroups (U.S. Food and Drug Administration, C.f.D.E.a.R., 1988). It was also mandated that new drug applications (NDAs) should include “effectiveness and safety data to be presented by sex and gender.

More decisive and broad guidelines regarding the inclusion of women were issued by the FDA in 1993, which reversed the previous restrictions for women participation in both Phase I and Phase II clinical trials (ADMINISTRATION, F.A.D., 1993) and instructed the drug sponsors to include both men and women in proportion reflecting the population who is the target for the therapeutic intervention (ADMINISTRATION, F.A.D., 1993; SERVICES, D.O.H.A.H., 1997). The NIH Revitalization Act (Public Law 103-43, 1993) codified these requirements, making the inclusion of women in NIH-funded research mandatory, prohibiting exclusion based on cost, and mandating outreach programs for recruitment (Congress, T.U., 1993). This reversal of prior restrictions was widely praised, though ethical and legal debates persisted regarding mandatory inclusion. This full reversal of previous restrictions was highly praised by the scientific community and women’s advocates as a major step forward in advancing women health, but debates continued to address ethical and legal aspects of the mandatory inclusion of women and the balance between inclusion and protection. A Committee on Ethical and Legal Issues Relating to the Inclusion of Women in Clinical Studies, Institute of Medicine (IOM), was tasked to address these concerns. The IOM committee noted that reversing the previous protections too abruptly and making women participation mandatory introduces potential risks, particularly in early-phase studies where reproductive and teratogenic risks are unknown (Mastroianni et al., 1994). To mitigate this concern without regressing to the paternalistic exclusion paradigm, the committee recommended a stepwise inclusion model, starting with participants at very low pregnancy risk after preclinical studies had been conducted (Mastroianni et al., 1994).

## Where we are today

The last few decades saw a strong increase in studies addressing sex differences in the development of cardiovascular disease (see review by *Laban* et al. in this collection describing the accumulation of knowledge regarding sex differences in the development of atherosclerosis (Laban et al., 2025). However, persistent challenges in representation of women within CV trials remain. Meta-analysis of 1, 079 cardiovascular trials registered on ClinicalTrials.gov from 2017 to 2023 and including 1, 396, 104 participants revealed that women were 41% of the participants and were specifically under-represented in trials for acute coronary syndrome and heart disease (32-39%) (Rivera et al., 2025). The representation of women in cardiac surgery trials was even lower: Only 20.8% of participants in cardiac surgery randomized controlled trials were women across 51 trials (Gaudino et al., 2021). Notably, the underrepresentation of women as participants was revealed to be correlated with the underrepresentation of women as principal investigators, suggesting that leadership directly impacts recruitment (Burgess et al., 2025). In addition to participant inclusion, the composition of research leadership may also influence how sex and gender are incorporated into biomedical research. Recent studies have shown that the presence of women as first or senior authors is associated with a higher likelihood of including sex-specific analyses and discussion of sex-related biological variables (Piani et al., 2024; Gulko et al., 2026). These findings suggest that representation in research leadership roles may shape the scientific questions being asked, the design of clinical trials, and ultimately the evidence base used to develop clinical guidelines. Increasing diversity in trial leadership and guideline committees may therefore represent an additional mechanism for improving the integration of sex-specific evidence into cardiovascular medicine.

Furthermore, beyond clinical trial design, a recent meta-analysis across 35 observational studies that included more than 13 million patients revealed that physician–patient sex concordance was typically associated with better outcomes, especially among female patients of female physicians (Heybati et al., 2025). These findings suggest that sex and gender influence not only participation in research and evidence generation, but also the delivery of care itself, further underscoring the importance of considering sex and gender across the entire continuum from research design to clinical practice.

The persistence of these disparities stems, at least in part, from the legacy of historical exclusion of women from clinical research. Early regulatory restrictions and entrenched research practices shaped study designs, recruitment strategies, and analytical approaches that did not prioritize sex-specific investigation. Although current policies mandate inclusion, many contemporary trials remain underpowered to detect sex differences, and sex-specific analyses are not consistently performed or reported. As a result, clinical guidelines are often based on evidence that does not fully account for biological sex and gender-related differences, requiring clinicians to extrapolate findings to female patients. This continued reliance on incomplete evidence contributes to ongoing challenges in the diagnosis, risk stratification, and treatment of cardiovascular disease in women.

Furthermore, the underrepresentation in medical research issue is not limited to women, but applies to other vulnerable populations, such as ethnic minorities and populations living in poverty, both experiencing barriers for participation based structural exclusion, digital divide, and trust deficit (Gehlert and Mozersky, 2018). This creates a cycle of underrepresentation in studies, leading to inaccurate findings, which subsequently further decreases trust and participation. More strategies are needed to address these barriers, including increase in women’s representation as principal investigators leading the studies.
